# The Land Gini Coefficient and Its Application for Land Use Structure Analysis in China

**DOI:** 10.1371/journal.pone.0076165

**Published:** 2013-10-09

**Authors:** Xinqi Zheng, Tian Xia, Xin Yang, Tao Yuan, Yecui Hu

**Affiliations:** 1 School of Land Science and Technology, China University of Geosciences (Beijing), Beijing, China; 2 Research Center for Operation and Development of Beijing, Institute of Policy and Management, Chinese Academy of Sciences, Beijing, China; Cinvestav-Merida, Mexico

## Abstract

We introduce the Gini coefficient to assess the rationality of land use structure. The rapid transformation of land use in China provides a typical case for land use structure analysis. In this study, a land Gini coefficient (LGC) analysis tool was developed. The land use structure rationality was analyzed and evaluated based on statistical data for China between 1996 and 2008. The results show: (1)The LGC of three major land use types–farmland, built-up land and unused land–was smaller when the four economic districts were considered as assessment units instead of the provinces. Therefore, the LGC is spatially dependent; if the calculation unit expands, then the LGC decreases, and this relationship does not change with time. Additionally, land use activities in different provinces of a single district differed greatly. (2) At the national level, the LGC of the three main land use types indicated that during the 13 years analyzed, the farmland and unused land were evenly distributed across China. However, the built-up land distribution was relatively or absolutely unequal and highlights the rapid urbanization in China. (3) Trends in the distribution of the three major land use types are very different. At the national level, when using a district as the calculation unit, the LGC of the three main land use types increased, and their distribution became increasingly concentrated. However, when a province was used as the calculation unit, the LGC of the farmland increased, while the LGC of the built-up and unused land decreased. These findings indicate that the distribution of the farmland became increasingly concentrated, while the built-up land and unused land became increasingly uniform. (4) The LGC analysis method of land use structure based on geographic information systems (GIS) is flexible and convenient.

## Introduction

Land use structure is a product of human activities and natural conditions. This structure consists of area proportion, space distribution and the influence of and relationship between all land use types. Land use structure indicates the status of natural and socioeconomic development; the structure affects (e.g., restricts) the development of all aspects of society. Land use structure rationality refers to the concept that the land use structure adapts to the harmony and sustainable development of society, the economy, and ecology. Land use structure optimization involves activities to organize the land use structure more rationally, which primarily includes area proportioning and space distributing. This optimization would increase the rationality of actual land use behavior and effectively balance various land use types, which promotes balanced land ecosystems. Furthermore, optimization facilitates the coordination and sustainable development of the economy, society, and ecology [1]. Analysis of land use structure rationality is the foundation of land use structure optimization. Land use structure is a well-established research field in land planning and land resources [2].

To understand the rationality of land use structure, the present status of the land use structure must be analyzed, and the problems of land use must be made explicit. The systematic theoretical research on urban land use structure was initiated in ecology in the 1920s. Subsequently, other schools of thought, such as economic location, social behavior, and political economics, were formed, and social science theories and varied analysis methods were developed. Classical models are presented in some of these theories. For example, the concentric circles model, the fan-shaped model, and the multi-core model are presented in the theory of economic location [3]. The research perspective has shifted from ecology to humanities (i.e., political and economic aspects) and has gradually turned to the functional space of urban areas [4], [5]. The research methods have shifted from traditional statistical analysis to information technology and multi-agent system modeling [6], [7]. Many scholars have researched land use structure in various regions [8] and at various spatial scales [13], [14]. These researchers have studied the status of one particular land use type [10], considered the eco-environment as the emphasis point of land use structure [15], analyzed land use structure on the basis of low carbon [13], used indices to understand the status of land use structure [12], or studied the order degree of land use structure [14]. As the methods, study regions, and emphases of the studies have not been consistent, the reported results have differed. Additionally, there is still no generally accepted method for assessing land use structure.

The scientific foundation, practicality and operability of analyses of land use structure rationality are critical to the viability of land use structure optimization plans and outcomes. Therefore, a suitable method to perform land use structure research should be chosen carefully. After generalizing and comparing the existing methods, it is apparent that some researchers have combined computer technology and mathematical theories to perform their studies. This technique allows for very rapid calculations and a variety of presentation options. Additionally, traditional geographical methods are still widely used and accepted because they are easy to understand and are well established. However, both methods have disadvantages. The former method can be overly complicated, and accurate programming is difficult. The latter method has analysis issues due to the lack of basic data and the unintuitive and limited methods for presenting results. Furthermore, neither of these two methods can quantify differences. Thus, the method for judging land use structure rationality needs to be improved. To combine the advantages of the methods described and to expand the research into land use structure, scholars have gradually introduced economic methods into studies of land use structure; the Gini coefficient model [16] is one of the most important of these methods [17].

Originally, the Gini coefficient was used in economics to assess the distribution of income. Recently, the coefficient was developed substantially for use in economics and applied more widely in other research fields [18], [19]. For example, this method has been applied to the distribution of household incomes under various conditions and impact assessments [20], [21], the distribution of medical resources [22], [23], the conditions and impact of plant growth [24]–[25], [26], and the consumption and use of material resources [27], [28]. The Gini coefficient method is also applied in environmental studies to understand water pollution issues and assist policy makers [29]–[30], [31]. These applications highlighted the universality of the Gini coefficient and popularized its use.

The Lorenz curve and Gini coefficient have been applied in land use analysis with encouraging results. The previous studies calculated numerical data, generated results and described the distribution of land use structure without considering spatial data. To improve the method for calculating the Gini coefficient, Yang (2008) explored dynamic calculation methods based on GIS spatial data. As a result, the calculation of the land Gini coefficient (LGC) was more operable and efficient [32].

Referencing previous research, this work applies the Gini coefficient to analyze land use structure and rationality. After defining the LGC, the paper combines the LGC with GIS, improves the LGC calculation tool, and builds a land use dataset from 1996 to 2008 for China. We calculate the LGC using various scales to analyze the spatiotemporal characteristics of the land use structure, evaluate the structure rationality, and gain new insights into land use in China.

## Materials and Methods

### Materials

We obtained land use data from 1996 to 2008 from the land use change survey managed by the land management department in China. This period was selected mainly because the statistical caliber and classification system of the data were consistent and the data were complete; the classification of land use types changed in 2009.

The population data are from the *Population Statistics of Counties in the People’s Republic of China from the Year 1997 to 2009* published by Masses Press [33]. Because the publication date was a year after the data collection date, the population data correspond to the same years as the land use data.

Based on the 1∶4,000,000 fundamental geographic information database, we built a dataset within the ArcGIS platform using the 13-year data by connecting the land use and population data with fundamental geographic information. This dataset contains the boundaries of cities, provinces and districts. The classification system used in this paper is that of the land use change survey during 1996 to 2008 [34]. This study analyzed the status of land use structure of the first level in the classification, which includes farmland, built-up land and unused land.

### Methods

#### The land gini coefficient

The major advantage of the Gini coefficient is that it can quantify differences, and it is very intuitive because it is based on the Lorenz curve. The Gini coefficient, originally used for quantifying differences in income, was reintroduced as the LGC to analyze the rationality of land use structure. The LGC has a different connotation for each target and has many sub-LGCs, called basic land use Gini coefficients, such as LGCAmount, LGCSpatial, and LGCMass.

The basic LGC involved in this study primarily includes LGCSpatial and LGCAmount. LGCSpatial indicates the evenness of a single land use type distribution in the study area, whereas LGCAmount indicates the rationality of the area proportions of all land use types in that area. The LGC ranges from 0 to 1, with smaller values indicating a more balanced land use structure. The standard for assessing different levels of land use structure based on the LGC is shown in Table 1.

**Table 1:** The standard to assess different levels of the LGC.

LGC	Less than 0.2	0.2–0.3	0.3–0.4	0.4–0.5	Greater than 0.5
Level	Absolutely equal	Relatively equal	Reasonable	Relatively unequal	Absolutely unequal

#### Calculation of the land gini coefficient

The main processes for calculating the LGC are constructing the land Lorenz curve, computing the basic LGC, and calculating the final LGC based on the basic LGC.

Because this study was mainly concerned with the distribution of land use in China, we illustrate the process for calculating LGCSpatial.

The steps of constructing the Traditional Lorenz Curve for LGCSpatial were following:

Step 1: Calculation of the location entropy of every land use type. The following formula is used:
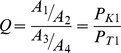
(1)where *Q* is location entropy, *A_1_* is the area of a certain land use type in a subordinate region of the calculation unit, *A_2_* is area of the same land use type of the calculation unit, *A_3_* is area of the subordinate region of the calculation unit, *A_4_* is area of the calculation unit, *P_K1_* is the percentage that *A_1_* occupies in *A_2_*, and *P_T1_* is the percentage that *A_3_* occupies in *A_4_*.

Step 2: Sorting the location entropy from small to large and calculating the cumulative percentages of the areas of each land use type and total area.

Step 3: Drawing the Lorenz curve of LGCSpatial based on the calculation and sorting results using the cumulative percentage of the total area as the X-coordinate and the cumulative percentage of each type of land use area as the Y-coordinate.

Demand and productivity of various land use types differ greatly and cannot be compared without transformation. The rational area of each land use type should be comprehensively determined with consideration of the ecological environment, the population that it serves, and the aims and directions of land use in the study area. Among those related factors, the population that a land use type serves could reflect the state of other factors; quantification of the population is the easiest and most viable of the factors. Thus, this study used the population served by a land use type to improve the index (i.e., location entropy). Location entropy is used for sorting in the construction of the traditional Lorenz Curve of LGCSpatial.

The steps of constructing the Improved Lorenz Curve of LGCSpatial were following:

Step 1: Calculation of the related index using the following formula:
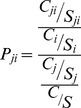
(2)where *P_ji_* is the related index of land use *i* in region *j*, *C_ji_* is the population that land use *i* serves in region *j*, *S_ji_* is the area of land use *i* in region *j*, *C_i_* is the total population that land use *i* serves in the entire study area, *S_i_* is the area of land use *i* in that area, *C_j_* is the population in region *j*, *S_j_* is the area of region *j*, *C* is the population of the entire study region, and *S* is its area. Region *j* is the subordinate study district of the entire study region.

Step 2: Sorting the index *P_ji_* from small to large and calculating the cumulative percentages of the areas of each land use type and the total area.

Step 3: Drawing the Lorenz curve of LGCSpatial based on calculations and sorting results using the cumulative percentage of the total area as the X-coordinate and the cumulative percentage of each type of land use area as the Y-coordinate.

The Gini coefficient calculation in GIS is based on a discrete distribution. Here, we used the method that Yang X et al [32]. proposed to improve and optimize the calculation of the basic LGC to make the calculation tool more suitable for this study.

The calculation process is as follows:

Step 1: The data are divided into different groups, the sum of each group is calculated, and the Lorenz curve is drawn as above.

Step 2: Because the connotations of the LGC and the basic LGCs are extended from the Gini coefficient in economics, and the formulas for calculating the basic LGCs and the interpretations of formulas are introduced from economics, the formula for basic LGC is as follows:
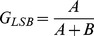
(3)where *G_LSB_* is LGCSpatial, *A* is the area between the data line and the diagonal (i.e., the perfect equality line), and *B* is the area between the data line and X-coordinate.

After translating formula (3), the formula to calculate *G_LSB_* is as follows:
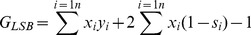
(4)where *n* is the code of group, *x_i_* is the percentage of subordinate study districts that the group shares with the entire study region sum, *y_i_* is the percentage of P-values that the sum of the group shares with the entire study region sum, and *s_i_* is the cumulative percentage of P-values quantified with the formula s_i_ = y_1_+ y_2_+ y_3_+……+ y_i_.

**Figure 1:**
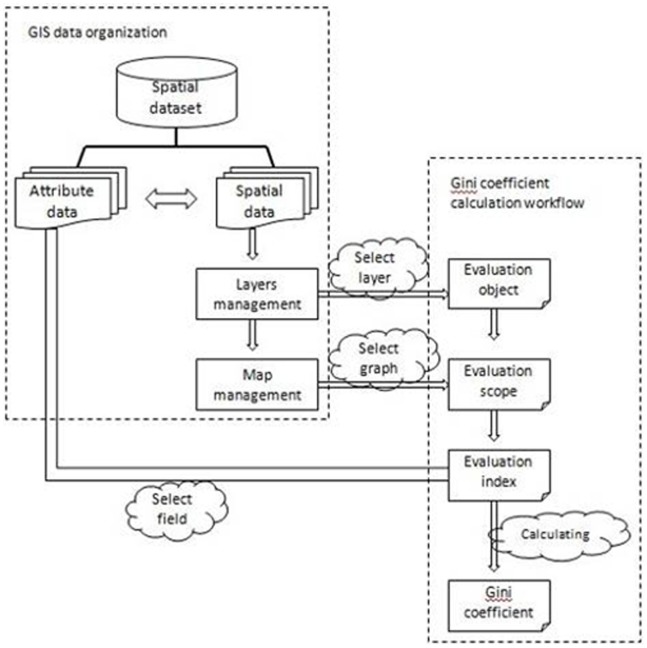
The dynamic calculation flow of the basic LGC based on GIS. Figure 1 illustrates the computing flow and the connection between the GIS data operations and the basic LGC calculation. It shows the dynamic selection of assessment units, dynamic adjustment of the assessment scope and dynamic selection of the assessment index.

**Figure 2:**
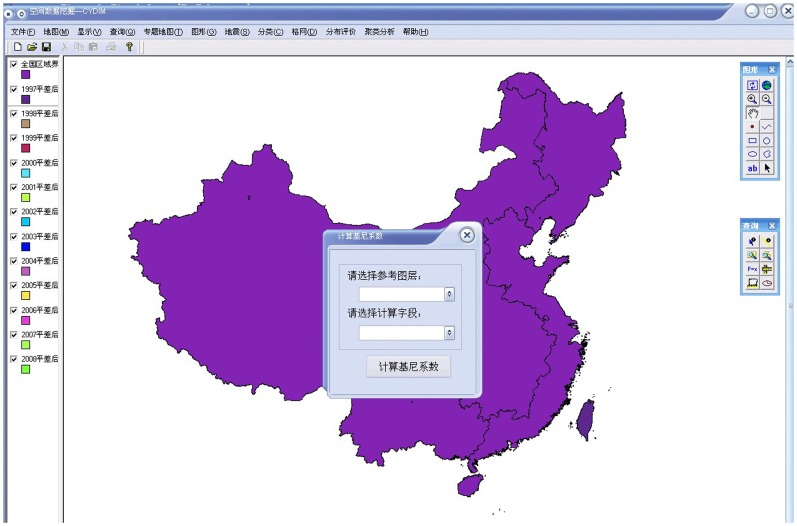
The operating interface of the LGC calculation tool. Figure 2 illustrates the operating interface of the LGC calculation tool. The visual graphical data operation renders the calculation of the basic LGC more intuitive and user-friendly. The data for calculating the basic LGC in GIS are the graphical data and all of the indices of each assessment unit at each level.

This calculation was developed into a tool using VC++ and MapObjects software. The tool can calculate the basic LGC dynamically, including the dynamic selection of assessment units, dynamic adjustment of the assessment scope and dynamic selection of the assessment index. This tool alters the traditional operation of the database and makes it possible to change the calculation objects by performing operations on the graphical data. The visual graphical data operation renders the calculation of the basic LGC more intuitive and user-friendly. The data for calculating the basic LGC in GIS are the graphical data and all of the indices of each assessment unit at each level. Figure 1 illustrates the computing flow and the connection between the GIS data operations and the basic LGC calculation. Figure 2 shows the operating interface of the LGC calculation tool.

Other basic LGCs can be calculated according to the method for calculating LGCSpatial. After all of the related basic LGCs are attained, the LGC can be calculated using a weighted sum method. To obtain the scientific LGC results, the weight of each basic LGC is determined from the impact of the basic LGCs on the LGC. The formula for this calculation is as follows:
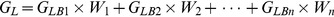
(5)where *G_L_* is the LGC, *G_LB1_* to *G_LBn_* are the basic LGCs, and *W_1_* to *W_n_* are the weights of each basic LGC. The weight of each basic LGC can be comprehensively determined by considering the suggestions from experts and the main issues regarding land use structure in China.

## Results and Discussions

After calculating and processing the data in ArcGIS 9.3 and using the aforementioned calculation tool, we obtained the results presented in the following sections.

### National Level

#### Results

In this paper, the study area was the Chinese mainland and Hainan Island. This area was divided into four regions–East, Northeast, Middle, and West–according to the four economic development districts. The Eastern district included 10 provinces: Beijing, Tianjin, Hebei, Shandong, Jiangsu, Shanghai, Zhejiang, Fujian, Guangdong, and Hainan. The Northeast district included three provinces: Heilongjiang, Jilin, and Liaoning. The West included twelve provinces based on the Western Development Strategy: Chongqing, Yunnan, Sichuan, Guizhou, Tibet, Guangxi, Xinjiang, Qinghai, Ningxia, Gansu, Shaanxi, and Inner Mongolia. The remaining provinces were in the Middle district [35].

LGCSpatial was calculated using districts and provinces as calculation units; the results are shown in Table 2.

**Table 2:** National LGCSpatial results using district and province calculation units.

Year	Calculation Unit	1996	1997	1998	1999	2000	2001	2002	2003	2004	2005	2006	2007	2008
Farmland	District	0.043	0.041	0.044	0.045	0.045	0.045	0.045	0.045	0.047	0.047	0.048	0.048	0.048
	Province	0.125	0.149	0.125	0.126	0.132	0.134	0.137	0.136	0.136	0.137	0.133	0.135	0.136
Built-up land	District	0.462	0.161	0.464	0.461	0.459	0.461	0.469	0.468	0.472	0.472	0.472	0.473	0.473
	Province	0.825	0.287	0.825	0.825	0.825	0.826	0.824	0.826	0.816	0.813	0.813	0.812	0.812
Unused land	District	0.127	0.173	0.136	0.136	0.13	0.132	0.133	0.134	0.135	0.137	0.144	0.145	0.145
	Province	0.249	0.341	0.28	0.281	0.283	0.283	0.281	0.281	0.275	0.276	0.277	0.278	0.278

As there are some mistakes in the data for 1997 and it is not viable to recalculate statistics, the results for 1997 are treated as noise and are excluded from the analyses.

#### Discussions

(1) Using a District as the Calculation Unit. The national LGCSpatial calculations using district units are presented in Table 2.

When four districts were used as the calculation units, the farmland and unused land were evenly distributed with LGCSpatial less than 0.2, but the distribution of the built-up land was relatively concentrated with LGCSpatial between 0.4 and 0.5. The trends in the distribution of the three land use types were very similar in that all became increasingly concentrated as LGCSpatial increased. The changes are reasonable, and the change in the farmland was less than the changes in the built-up and unused land. This finding indicates that the farmland decreased at the expense of the occupied built-up land and from destruction caused by disasters, among other activities. Although many activities have been put into practice to supplement farmland, the trend towards the increasingly concentrated distribution of farmland has not ceased. Nevertheless, stringent government policies protecting farmland, especially cultivated land, have had significant results. For example, the government aims to protect farmland as “18 million hectares of cultivated land” and protect capital farmland. This policy has ensured that the quantity of farmland has not decreased below 18 million hectares and that the quality of farmland (particularly cultivated land) improved over the past 13 years. LGCSpatial reflects the effect of these policies on slow growth. In the 13 years analyzed, considerable manpower and materials have been invested in reclamation projects and development of unused land, such as the Gorges Reservoir Area Fertilizing Project in Chongqing. Such projects have put extensive unused land into rational use and have led to the reutilization of abandoned land. These activities have reduced the amount of unused land and increased its concentration, as indicated by the increasing LGCSpatial statistic. The remaining unused land is difficult to develop into usable land. The trend toward the concentration of built-up land reflects economic development in China. Chinese development strategies include using cities to help rural areas develop and improving the agricultural modernization process (i.e., industrialization). These factors have led to rapid economic improvement and urbanization in the East District. In this region, the area of built-up land has increased greatly, and this land has had an obvious agglomeration effect in rapidly developing districts.

(2) Using a Province as the Calculation Unit.

The national LGCSpatial values using provinces as the calculation unit are shown in Table 2. During the 13 years analyzed, the farmland was very evenly distributed across the country with LGCSpatial less than 0.2, and the distribution of unused land was relatively equal with LGCSpatial between 0.2 and 0.3. However, the distribution of the built-up land was absolutely unequal with LGCSpatial greater than 0.8. The trends in distribution vary with land use types. The farmland became increasingly concentrated as LGCSpatial increased, while the built-up land and unused land became more equal as LGCSpatial decreased. The built-up land changed the quickest, whereas the unused land changed slowly. These results indicate that at the beginning of the study period, the percentages of farmland area were approximately the same in most provinces, whereas the percentages of built-up land area varied greatly (e.g., in the Zhejiang Province). Meanwhile, the farmland was much more abundant than the built-up land, but the percentage of built-up land increased while the farmland decreased. These changes caused the evenly distributed farmland to become increasingly concentrated, whereas the built-up land spread out. However, the distribution of built-up land remained absolutely unequal. Adjustment of the structure and distribution of the built-up land will be a major problem requiring government attention. Some provinces, such as Gansu Province, had greater proportions of unused land that could be developed, excluding snow mountains; the development is associated with greater comprehensive benefits compared to other land use types. Furthermore, these provinces performed more research on developing the unused land and used more advanced technology compared with other provinces. This difference led to the uneven distribution of development. The provinces with greater areas of unused land developed more than the other provinces, which evened out the distribution of unused land.

(3) Comparison of Results Using Different Calculation Units.

Comparing the results using the different calculation units listed in Table 2, we observe that LGCSpatial values calculated using province units are larger than those using district units. Therefore, LGCSpatial is higher when the area of the calculation unit is smaller. However, the principal situation and the resultant trend are essentially the same regardless of the calculation unit. This finding shows that LGCSpatial is inversely affected by scale (i.e., it decreases with a larger calculation unit). Comparing these results also reveals that land use activities differ greatly in individual provinces within a district and are more apparent with built-up land and unused land. Therefore, individual provinces within a district are at varying developmental stages and conditions.

Additionally, the results using district units show that the three main land use types are becoming increasingly concentrated. However, when province units were used, the farmland became more concentrated, whereas the built-up land and unused land were reduced. This finding indicates that the development speed is not balanced between the districts. A district at an advanced developmental stage is developing faster. The development speed in a district is balanced between the individual provinces. The underdeveloped provinces develop slightly faster than the provinces at advanced development stages within a district. This observation indicates that the regional economic development strategy to develop the East and Northeast Districts first, followed by the West District, and the Middle District last is well-implemented.

### District Level

#### Results

**Figure 3:**
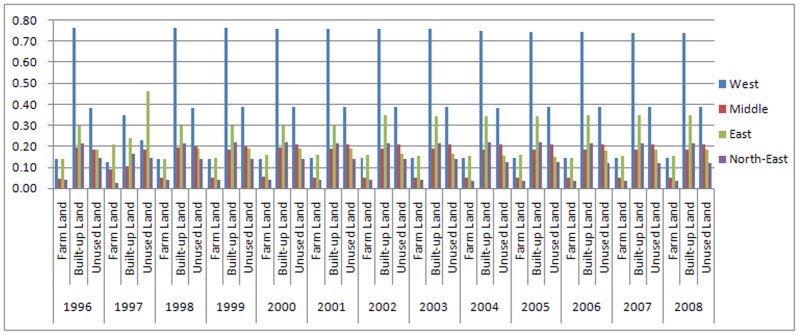
District LGCSpatial in province units. Figure 3 is the LGCSpatial of the three main land use types for each study year in the four districts using province units at the district level.

**Figure 4:**
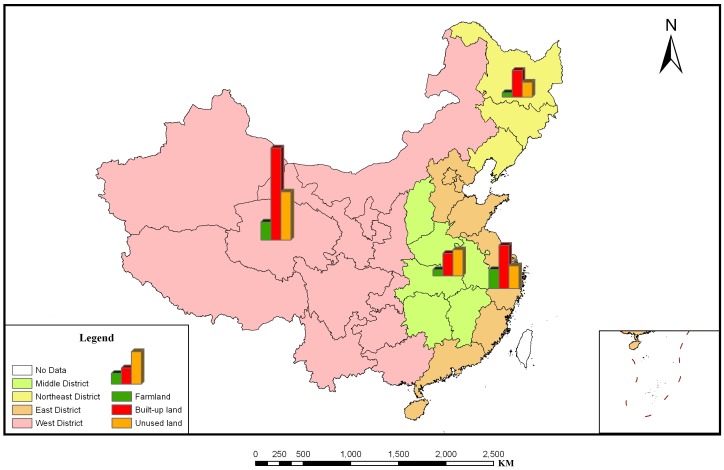
District LGCSpatial of the three main land use types in 2008. Figure 4 depicts a sample of the results from 2008, which demonstrates that the LGC can be visualized and portrayed using the ArcGIS platform.

After performing calculations using province units at the district level, we obtained LGCSpatial of the three main land use types for each study year in the four districts (Figure 3). Figure 4 depicts a sample of the results from 2008, which demonstrates that the LGC can be visualized and portrayed using the ArcGIS platform.

As there are some mistakes in the data for 1997 and it is not viable to recalculate statistics, the results for 1997 are treated as noise and are excluded from analyses.

#### Analysis and Discussions

The district LGCSpatial values (in province units) reveal that the distributions and trends of the three land use types are very different at the district level.

**Figure 5:**
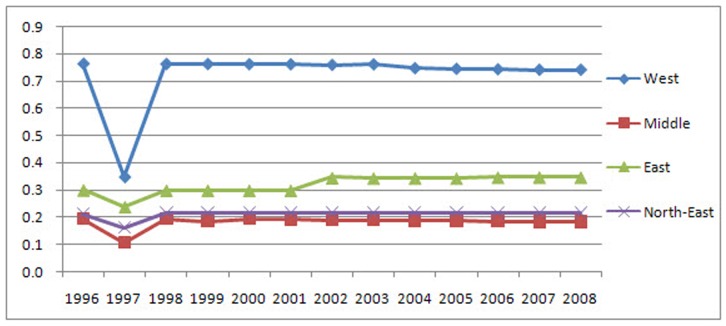
The district LGCSpatial values and associated trends of built-up land. The results indicate that the LGCSpatial values of built-up land in the West District are larger than 0.70; therefore, the distribution of built-up land in this region is completely unequal. In the East, these values are between 0.29 and 0.35, indicating that the distribution of built-up land is reasonable. In the Northeast, the LGCSpatial values are between 0.20 and 0.22, which indicate that the distribution of this type of land is relatively equal. In the Middle District, the values are less than 0.2, demonstrating that the distribution is absolutely equal. The trends varied across districts. The built-up land is increasingly concentrated in the East with an approximate 0.05 increase in LGCSpatial, more evenly distributed in the Middle and West Districts with approximate decreases in LGCSpatial of 0.01 and 0.02, respectively, and stable in the Northeast.

The results indicate that the LGCSpatial values of built-up land in the West District are larger than 0.70 (Figure 5); therefore, the distribution of built-up land in this region is completely unequal. In the East, these values are between 0.29 and 0.35, indicating that the distribution of built-up land is reasonable. In the Northeast, the LGCSpatial values are between 0.20 and 0.22, which indicate that the distribution of this type of land is relatively equal. In the Middle District, the values are less than 0.2, demonstrating that the distribution is absolutely equal. The trends varied across districts. The built-up land is increasingly concentrated in the East with an approximate 0.05 increase in LGCSpatial, more evenly distributed in the Middle and West Districts with approximate decreases in LGCSpatial of 0.01 and 0.02, respectively, and stable in the Northeast.

**Figure 6:**
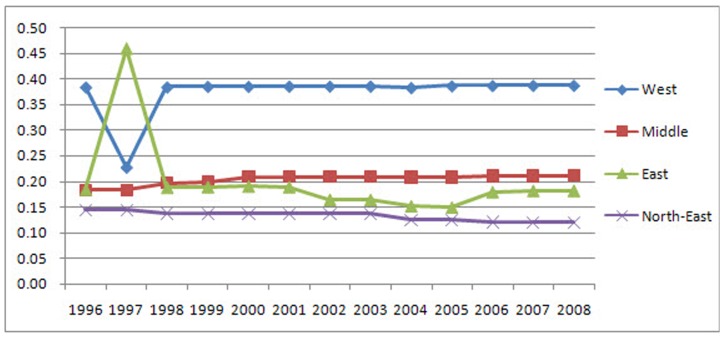
The district LGCSpatial values and trends of the unused land. The LGCSpatial values of unused land in the West District are between 0.3 and 0.4, indicating that the distribution of unused land is reasonable. In the Middle District, the values are between 0.18 and 0.22, suggesting that the distribution of unused land is relatively equal. In the East and Northeast Districts, the values are less than 0.2, indicating an absolutely equal distribution. The trend observed in the East District is unique, as it repeatedly changed: first tending to disperse, then returning to the original level with an approximate LGCSpatial change of 0.03. The Middle District is becoming increasingly concentrated with a LGCSpatial increase of approximately 0.03. The Northeast District is becoming less concentrated with a LGCSpatial decrease of approximately 0.02. The concentration of the distribution of unused land in the West District has slightly increased.

As observed in Figure 6, the LGCSpatial values of unused land in the West District are between 0.3 and 0.4, indicating that the distribution of unused land is reasonable. In the Middle District, the values are between 0.18 and 0.22, suggesting that the distribution of unused land is relatively equal. In the East and Northeast Districts, the values are less than 0.2, indicating an absolutely equal distribution. The trend observed in the East District is unique, as it repeatedly changed: first tending to disperse, then returning to the original level with an approximate LGCSpatial change of 0.03. The Middle District is becoming increasingly concentrated with a LGCSpatial increase of approximately 0.03. The Northeast District is becoming less concentrated with a LGCSpatial decrease of approximately 0.02. The concentration of the distribution of unused land in the West District has slightly increased.

**Figure 7:**
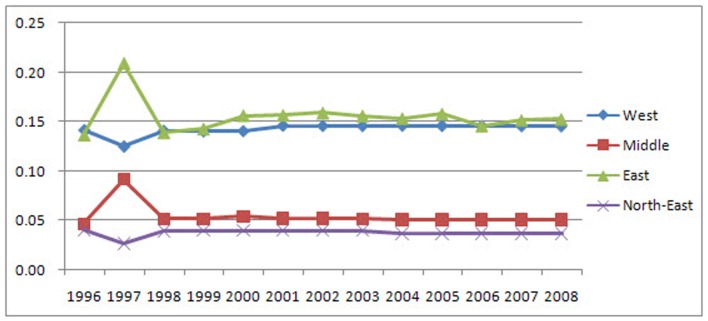
The district LGCSpatial values and trends of the farmland. The distributions and trends of farmland are different than those of built-up and unused land. The LGCSpatial values of farmland are less than 0.2 in every district, indicating that farmland is absolutely equally distributed in all districts. In the Northeast, Middle and West Districts, farmland remained virtually stable, while in the East, it remained stable with slight fluctuations.

As observed in Figure 7, the distributions and trends of farmland are different than those of built-up and unused land. The LGCSpatial values of farmland are less than 0.2 in every district, indicating that farmland is absolutely equally distributed in all districts. In the Northeast, Middle and West Districts, farmland remained virtually stable, while in the East, it remained stable with slight fluctuations.

The most important reasons for these distributions are the environmental conditions and population distributions, but the economy and policies also play roles.

Concerning built-up land, the West District is associated with a larger area, smaller population and worse eco-environmental conditions than other districts; the population and suitable areas for built-up land are very concentrated, and the need for built-up land is lower than that in other districts (thus, any built-up land in the West District would be concentrated). However, the associated LGCSpatial value is larger than 0.70 (i.e., absolutely unequal); the excessive concentration does not support sustainable development and requires improvement. The most developed district is the East. Because the built-up land has an agglomeration effect, the distribution of this type of land is reasonable. The policy is to develop the East and West first and the Middle District last. The development of the Northeast Dis faster and more stable than that of the Middle District. The mountains and hills of the Middle District, unlike the other districts, make it difficult to concentrate built-up land. The development of the West District is not sufficient to cause an agglomeration effect of the built-up land. Overall, these factors have caused the observed trends in built-up land.

Within farmland, crop types depend on local geography, climate and other eco-environmental conditions; methods for crop production meet local needs. The production of all crops in each district is essential; therefore, the distributions and trends of farmland are inherently rational. The East is the most developed district, the West has the most severe eco-environment conditions, and the Middle and Northeast Districts have moderate development; it is reasonable that the LGCSpatial values of the East and West Districts are higher than those of the Middle and Northeast Districts.

Many types of unused land, such as snow mountains, are mainly centralized and distributed throughout the West District, whereas extreme terrain is sparse in other districts. Therefore, the distribution of unused land in the West District is more concentrated, along with larger LGCSpatial values. Given the limitations imposed by the eco-environmental conditions, technology, and the shortage of economic development, the development of unused land is difficult to implement in the West. Because only a small area of unused land is developed, its distribution in the West is slightly centralized. The distribution and trend of unused land in the East are mainly influenced by economic development. As the East District is the most developed and the eco-environmental conditions are conducive for developing unused land, developing large areas of this land has an agglomeration effect. Therefore, development tends to disperse, and the LGCSpatial value decreases with the development of large areas of unused land in the East District, which is eventually utilized in the first stage. The remaining unused land is smaller in scale, and its development causes an increasingly centralized distribution with an increasing LGCSpatial value. In the Middle and Northeast Districts, the distributions and trends of unused land are determined by eco-environmental and economic factors.

### Inspiration of LGC for Improving the Land Use Spatial Structure

The LGCSpatial results indicate that the built-up land is sufficiently concentrated. We suggest that the government take the necessary steps to stop or slow down the transformation of built-up land distribution. Built-up land, at a certain centralization level, is beneficial for social development, but if the distribution is too concentrated, then it has a negative effect on society.

The distribution of farmland should remain stable considering its present concentration. Farmland production varies by region and local conditions; thus, the LGCSpatial values of farmland should be at an absolutely equal level (i.e., LGCSpatial less than 0.2).

The distribution of unused land should remain unchanged or become slightly concentrated to maintain reasonable proportions and ensure its protection.

## Conclusions

This paper presents the concept of the LGC and basic LGCs. The GIS-based LGC calculation tool can efficiently calculate the LGC and visualize the results for simplified comprehension. We calculated and analyzed land use data from 1996 to 2008 using two different calculation units for various land use types in China. We discerned the temporal and spatial changes of LGCSpatial values and analyzed the associated policies and economic backgrounds. Based on this research, suggestions on land use structure optimization using the LGC were presented. The technology and methodology reported can help to analyze and optimize land use structure, including spatial distribution and temporal arrangement. This work indicates that it is necessary to incorporate the Gini coefficient into land use research and that this coefficient will be helpful in related research.

We have also shown that LGCSpatial values are affected by scale. Use of differing calculation units yields varying LGCSpatial results. The LGCSpatial values increase when the scale of the calculation unit decreases.

Based on our method and calculation tool using the LGC to analyze land use structure, cities and counties can be used as calculation units in research. This method can be used to analyze the distribution of each land use type at a smaller scale and elicit more specific and effective suggestions for optimizing land use structure. We could also calculate and examine the LGC of the first, second and third levels of land use types, rather than the first level only, to optimize the land use structure (i.e., more detailed data). Boundaries are very important for analyzing the results and should be varied based on the calculation unit; future research may consider this aspect. Additionally, research on other basic LGCs will gradually be put into practice to improve this method and calculation tool.
